# Long Non-Coding RNA LINC00467 Correlates to Poor Prognosis and Aggressiveness of Breast Cancer

**DOI:** 10.3389/fonc.2021.643394

**Published:** 2021-04-28

**Authors:** Ying Zhang, Yi Sun, Lin Ding, Wenjing Shi, Keshuo Ding, Yong Zhu

**Affiliations:** ^1^ Department of Pathophysiology, School of Basic Medical Sciences, Anhui Medical University, Hefei, China; ^2^ Department of Oncology of the First Affiliated Hospital, Division of Life Science and Medicine, The CAS Key Laboratory of Innate Immunity and Chronic Disease, University of Science and Technology of China, Hefei, China; ^3^ Department of Naval Medicine, Naval Medical University, Shanghai, China; ^4^ Department of Pathology, Anhui Medical University, Hefei, China

**Keywords:** breast cancer, EMT, LINC00467, miR-138-5p, LIN28B

## Abstract

Breast cancer remains the leading cause of female cancer-related mortalities worldwide. Long non-coding RNAs (LncRNAs) have been increasingly reported to play pivotal roles in tumorigenesis and cancer progression. Herein, we focused on LINC00467, which has never been studied in breast cancer. Silence of LINC00467 suppressed proliferation, migration, invasion and epithelial-to-mesenchymal transition (EMT) of breast cancer cells *in vitro*, whereas forced expression of LINC00467 exhibited the opposite effects. Furthermore, we demonstrated overexpression of LINC00467 promoted tumor growth, while knockdown of LINC00467 inhibited pulmonary metastasis *in vivo.* Mechanistically, LINC00467 down-regulated miR-138-5p by acting as a miRNA “sponge”. Besides, LINC00467 also up-regulated the protein level of lin-28 homolog B (LIN28B) *via* a direct interaction. A higher expression level of LINC00467 was observed in breast cancer tissues as compared to the adjacent normal counterparts and elevated LINC00467 predicted poor overall survival. Our findings suggest LINC00467 promotes progression of breast cancer through interacting with miR-138-5p and LIN28B directly. LINC00467 may serve as a potential candidate for the diagnosis and treatment of breast cancer.

## Introduction

Breast cancer is the most prevailing type of female malignancy, leading cancer-related death of women globally (1). Surgical removal and chemotherapy are still the mainstay of clinical management options (2). However, the prognosis of breast cancer patients remains unsatisfactory due to the local recurrence or distant metastasis (3). It is therefore urgent to conduct in-depth research about the molecular mechanisms underlying tumor growth and metastasis for development of new effective diagnosis and treatment for breast cancer patients.

Long noncoding RNAs (lncRNAs), a class of RNA molecules longer than 200 nucleotides, with less protein-coding capacity, have been reported to play crucial roles in multiple cellular processes, tumor progression included (4–6). In general, LncRNAs function *via* interacting with proteins, chromatin DNA or other types of RNAs to mediate their stability or activity in different aspects of tumor biology (7–10).

LINC00467 (ID in NCBI database: 84791), a LncRNA has attracted much attention recently, was reported to be dysregulated in spermatogenesis and male infertility (11, 12). Besides, it was also reported that LINC00467 expression was associated with Vogt-Koyanagi-Harada Disease and Behcet’s Disease (13). Numerous studies indicated LINC00467 acted as an oncogene in colorectal cancer, lung adenocarcinoma, hepatocellular carcinoma, glioma, head and neck squamous cell carcinoma and acute myeloid leukemia (14–19). Nevertheless, the expression pattern and functional role of LINC00467 in breast cancer has been less explored.

Here, we verified that LINC00467 promoted proliferation, migration, invasion and EMT of breast cancer cells both *in vitro* and *in vivo*. We further demonstrated LINC00467 inhibited the expression of miR-138-5p and up-regulated the protein level of LIN28B *via* binding to each other directly. Expression level of LINC00467 was higher in breast cancer cells and tissues as compared with normal counterparts, respectively. High level of LINC00467 was positively associated with poor prognosis of breast cancer patients. These results offer new mechanistic insight into breast cancer progression and suggest a potential therapeutic value of LINC00467.

## Materials and Methods

### Cell Lines

SKBR-3, MCF-7, T47D, MDA-MB-231, and BT-549 were purchased from the American Type Culture Collection (Rockville, MD, USA), and cultured as recommended. HMEC-hTERT cell line was a kind gift from Prof. William Hahn’s lab and cultured as previously described (20). SUM-149 cells was obtained from Asterand (21) and cultured according to the same protocol (9).

### Manipulation of Gene Expression

LINC00467 overexpression cell lines were established by infection of lentivirus containing full-length LINC00467-inserted pSin vector. Lentivirus containing shRNAs against LINC00467 was produced to knockdown the expression of LINC00467. Detailed protocols of lentivirus production and stable cell line construction were described previously (22, 23). Transfection of miRNA mimics was performed using Lipofectamine 2000 (Invitrogen, MA, USA) according to the instruction. Oligonucleotides used were listed in **Supplementary Table 1**.

### RNA Extraction and qRT-PCR

Total RNA of indicated cells and tissues was isolated with Trizol (Invitrogen, Carlsbad, CA), cDNA was prepared by reverse transcription of mRNA and miRNA using RevertAid First Strand cDNA Synthesis Kit (Thermo Scientific Bio) accordingly to the manufacturer’s manual, the resulting cDNA samples were analyzed by quantitative real-time PCR using a PerfectStart™ Green qPCR SuperMix (TransGen) on a CFX96 Touch Real-Time PCR Detection System (Bio-Rad, CA, USA). All primers used were listed in **Supplementary Table 1**.

### Cell Functional Assays

To assess cell proliferation, MTT assay and colony formation assay were performed as described in the previous study (9). EdU cell viability assay was conducted as recommended (cat. no. KGA331-100; Jiansu KeyGen BioTECH Co. Ltd). Briefly, 10^4^ stably transfected breast cancer cells were plated in 96-well plates for 24 h and then labeled with EdU for 4 h, cells were then fixed with 4% paraformaldehyde and washed with PBS. Cell nucleus was stained with hoechst-33342 at room temperature for 15 min. Images of stained cells were captured by using ZEISS AXIO fluorescence microscope (magnification, x20).

Cell migration and invasion were determined by Wound-healing assay and Transwell assay as described previously (9, 24).

### Protein Extraction and Western Blot

Total protein from indicated cells was extracted with the modified RIPA lysis buffer. Western blot was performed as we previously described (25), and imaged with ECL detection system (Bio-Rad, CA, USA). The primary antibodies from Proteintech used were anti-E-cadherin (20874-1-AP), anti-N-cadherin (22018-1-AP), anti-MMP9 (10375-2-AP), anti-Vimentin (10366-1-AP), anti-β-actin (66009-1-Ig) and anti-LIN28B (16178-1-AP).

### Xenograft and Histological Analysis

For tumor growth assay, 5×10^6^ LINC00467-overexpressing MCF-7 cells and the corresponding control mixed 1:1 with Matrigel (BD Biosciences) were injected into the second mammary fat pad of female Balb/c nude mice (5 weeks old) which were injected intraperitoneally with estradiol pellets (Innovative Research of America) a week ago. The tumor volumes were calculated with the equation: width^2^ × length × 0.5. At day 28, mice were sacrificed and tumors were resected for Ki-67 staining (24).

For lung metastasis assay, 2×10^6^ LINC00467-knockdown MDA-MB-231 cells and the corresponding control were injected intravenously (tail vein) into female Balb/c nude mice. Lungs were harvested about two months later and subjected to hematoxylin and eosin (H&E) staining.

All animal experiments were approved by the Animal Ethical Committee of Anhui Medical University.

### Luciferase Reporter Assays

Full length of wide-type LINC00467 was inserted into luciferase reporter vector psiCHECK-2, the mutant LINC00467 psiCHECK-2 vector was amplified by using the special primers according to the wide-type LINC00467 psiCHECK-2 vector. All primers used were listed in **Supplementary Table 1**. The detailed protocol of luciferase reporter assays was described in our previously study (9).

### Biotin RNA Pull-Down Assay and RNA Immunoprecipitation Assay

The detailed protocol was described previously (9, 26), the sense or antisense biotin-labeled DNA oligomers against LINC00467 were listed in **Supplementary Table 1**. As the RNA immunoprecipitation assay, anti-IgG (Santa Cruz, sc-2025), anti-LIN28B (Santa Cruz, sc-374460), and the Protein A/G agarose (Santa Cruz, sc-2003) were used as recommended, the immunoprecipitated RNA was converted to cDNA for further qRT-PCR analysis.

### Clinical Specimens

A total of 70 breast cancer tissues with their paired adjacent normal mammary tissues were collected from the First Affiliated Hospital of Anhui Medical University (Hefei, Anhui, People’s Republic of China), and verified by at least three experienced pathologists independently. All patients enrolled signed their informed consent for the research. No study was initiated before all clinical research protocol was approved by the Biomedical Ethics Committee of Anhui Medical University which was in accordance with the Declaration of Helsinki.

### Statistical Analysis

GraphPad Prism 8.0 was used for data processing and analysis. Data are presented as mean ± standard deviation (SD). Student’s t-test, two-way ANOVA, chi-squared test and Kaplane-Meier’s analysis were employed for the proper statistical comparisons. All experiments were repeated least three times and P < 0.05 was regarded as statistically significant.

## Results

### LINC00467 Promoted Proliferation of Breast Cancer Cells

**Figure 1:**
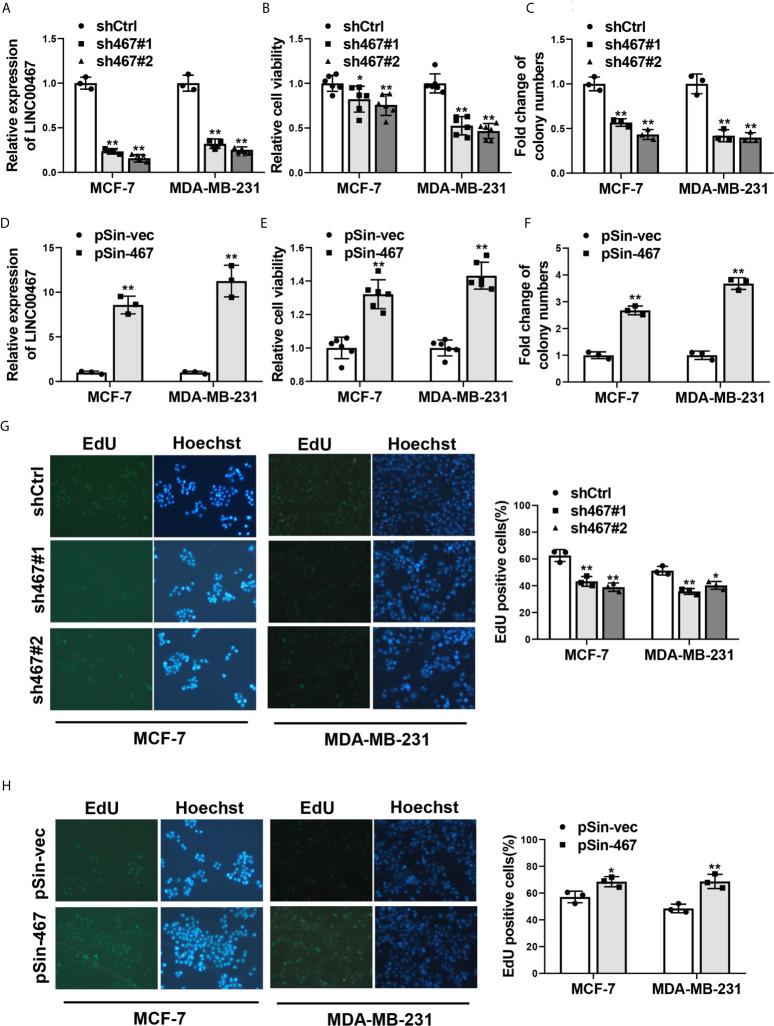
Effects of LINC00467 on proliferation of breast cancer cells. **(A)** qRT-PCR analysis of LINC00467 expression in MCF-7 and MDA-MB-231 cells stably transfected with two specific shRNAs against LINC00467 (sh467#1 and sh467#1) or pLKO.1 empty vector (shCtrl). **(B, C)** Cell proliferation of LINC00467-silenced MCF-7 and MDA-MB-231 cells was determined by MTT assay and colony formation assay. **(D)** LINC00467 expression in MCF-7 and MDA-MB-231 cells stably transfected with pSin plasmid harboring LINC00467 sequence (pSin-467) or pSin empty vector (pSin-vec) was examined by qRT-PCR. **(E, F)** Cell proliferation of MCF-7 and MDA-MB-231 cells with LINC00467 overexpression was determined by MTT assay and colony formation assay. **(G, H)** EdU incorporation assay in MCF-7 and MDA-MB-231 cells with LINC00467 depletion **(G)** or overexpression **(H)**. Images were taken at 200× magnification. Data are presented as mean ± S.D from three independent experiments. *P < 0.05; **P < 0.01. Student’s t-test.

To assess the role of LINC00467 in aggressiveness of breast cancer, we first determined if LINC00467 mediated malignant phenotype of breast cancer cells. Two most representative breast cancer cell lines: MCF-7 and MDA-MB-231, were chosen for functional verification. MCF-7 and MDA-MB-231 cells were stably transfected shRNAs against LINC00467 *via* lentivirus infection to knockdown the endogenous expression of LINC00467, the knockdown efficiency was confirmed by qRT-PCR (**Figure 1A**). Cell viability and colony-formation capacity were significantly inhibited with the silence of LINC00467, as determined by MTT assay (**Figure 1B**) and colony formation assay (**Figure 1C**). On the other hand, we complemented MCF-7 and MDA-MB-231 cells with ectopic LINC00467 to generate stable overexpression of LINC00467 (**Figure 1D**). Remarkably enhanced cell viability and colony-formation capacity were observed in LINC00467-overexpressing breast cancer cells in comparison with the control ones (**Figures 1E, F**). Besides, EdU incorporation assay showed depleted LINC00467expression repressed, while forced expression of LINC00467 increased proliferation of both MCF-7 and MDA-MB-231 cells (**Figures 1G, H**). Taken together, these results suggested LINC00467 was able to significantly enhance proliferation of breast cancer cells.

### LINC00467 Enhanced Migration, Invasion and EMT in Breast Cancer Cells

**Figure 2:**
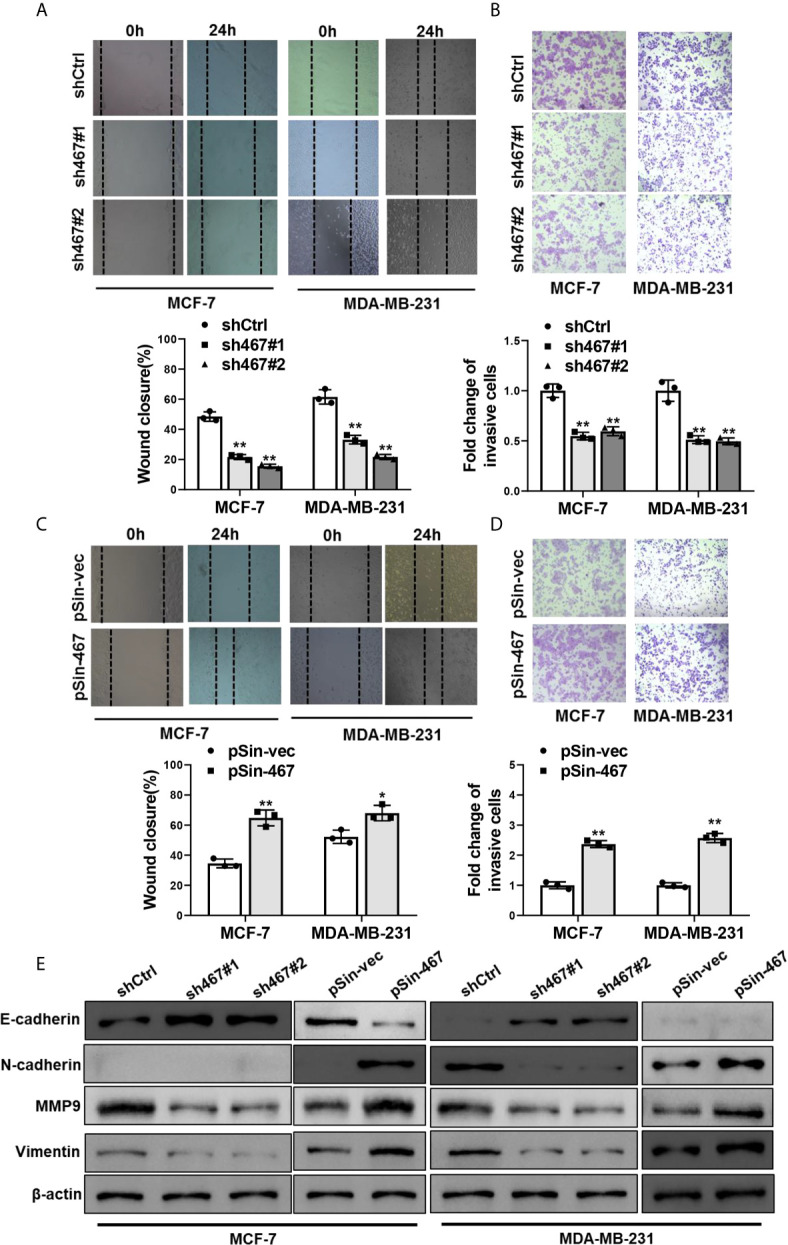
Manipulation of LINC00467 expression mediated migration, invasion and EMT of breast cancer cells. **(A, B)** Migratory and invasive capacities of LINC00467-depleted MCF-7 and MDA-MB-231 cells were assessed by Wound healing assay and Transwell assay, respectively. **(C, D)** Wound healing assay and Transwell assay were performed to assess migratory and invasive capacities of LINC00467-overexpressing MCF-7 and MDA-MB-231 cells. **(E)** Protein levels of EMT markers in MCF-7 and MDA-MB-231 cells with LINC00467 knockdown or overexpression were determined by Western blot. Images were taken at 100× magnification. Data are presented as mean ± S.D from three independent experiments. *P < 0.05; **P < 0.01. Student’s t-test.

Next, we examined the effects of manipulating the expression of LINC00467 on migration and invasion in MCF-7 and MDA-MB-231 cells. Similarly, suppressing LINC00467 expression reduced, whereas ectopic expression of LINC00467 increased migratory and invasive capacity as compared with the corresponding control ones (**Figures 2A–D**). Epithelial-to-mesenchymal transition plays a pivotal role in metabolic reprogramming (27), chemoresistance (28), and stemness maintenance (29), especially cell mobility and tumor metastasis in breast cancer (30, 31). Therefore, we sought to explore whether LINC00467 mediated EMT progress or not. As expected, depletion of LINC00467 led to upregulation of epithelial marker (E-cadherin) and inhibition of mesenchymal markers (N-cadherin, MMP9 and Vimentin). Oppositely, overexpression of LINC00467 resulted in increased expression of mesenchymal markers (N-cadherin, MMP9 and Vimentin) and decreased expression of epithelial marker (E-cadherin) (**Figure 2E**). Collectively, these results indicated that LINC00467 promoted migration, invasion and EMT of breast cancer cells.

### LINC00467 Promoted Tumor Growth and Lung Metastasis of Breast Cancer Cells in Nude Mice

**Figure 3:**
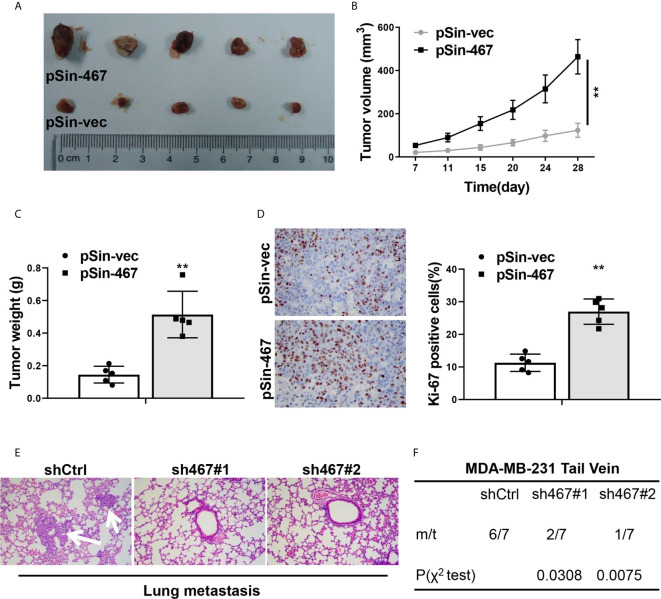
LINC00467 promoted progression of breast cancer cells *in vivo*. **(A)** Images of tumor nodules derived from LINC00467-overexpressing MCF-7 cells (pSin-467) or the corresponding negative control ones (pSin-vec). **(B)** Tumor volumes of each group were measured at the indicated days. **(C)** Tumors were resected and weighted after the last measurement at day 28. **(D)** Ki-67 expression of tumor sections from each group was determined by immunohistochemistry. Images were taken at 400× magnification. **(E, F)** MDA-MB-231 cells with LINC00467 knockdown (sh467#1 and sh467#1) or control (shCtrl) were injected intravenously into nude mice via tail vein. H&E staining of the metastatic nodules in the lungs of each group, which were indicated by white arrows. Images were taken at 200× magnification **(E)** and incidence of lung metastasis of each group **(F)**. **P < 0.01. (Two-way ANOVA for Panel B, χ^2^ test for Panel F, Student’s t-test for others).

To further determine the oncogenic role of LINC00467 *in vivo*, the xenograft tumor model was employed to evaluate tumor growth derived from MCF-7 cells with LINC00467 expression manipulation. In consistent with the results *in vitro*, LINC00467-overexpressing-MCF-7 cells formed clearly larger tumors than MCF-7-vector cells did, tumor growth curve showed forced expression of LINC00467 led to a significantly faster growth rate (**Figures 3A–C**). Ki-67 expression of tumor sections from both groups was detected by immunohistochemical staining, it came out that there was an obviously higher proportion of proliferative cells in LINC00467-overexpressing-MCF-7-derived tumors compared with MCF-7-vector-derived tumors (**Figure 3D**). We then assessed the effects of LINC00467 on lung metastasis, MDA-MB-231 cells stably transfected with either shRNAs targeting LINC00467 or empty vector, were transplanted into nude mice by tail vein injection. About 8 weeks later, lung tissues of each group were harvested to determine the incidence of lung metastasis. H&E staining of lung sections revealed the number and size of micrometastases remarkably reduced in lungs of mice intravenously injected with LINC00467 ablation MDA-MB-231 cells comparing with those of shCtrl mice (**Figure 3E**). Besides, a much lower frequency of lung metastases was observed in mice injected with MDA-MB-231-sh467 cells (**Figure 3F**). Taken together, all these results suggested LINC00467 acted as a crucial role in breast cancer growth and metastasis.

### LINC00467 Acted as a “Sponge” of miR-138-5p in Breast Cancer Cells

**Figure 4:**
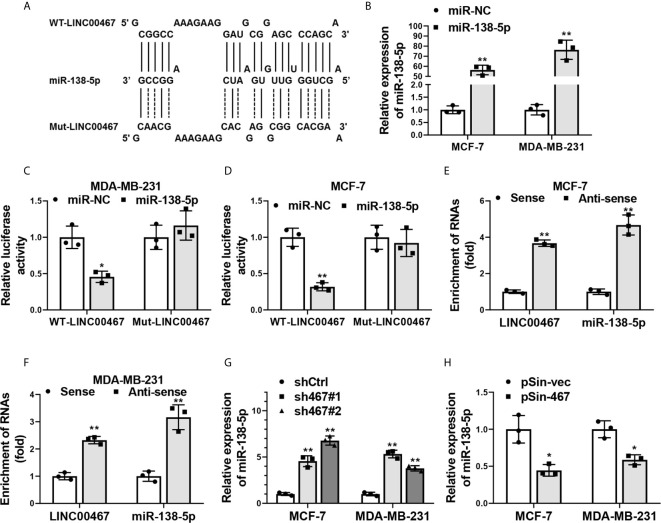
LINC00467 was physically associated with miR-138-5p. **(A)** Schematic outlining the predicted wild-type (WT-LINC00467) or mutant (Mut-LINC00467) binding sites of miR-138-5p on LINC00467 transcripts. **(B)** Overexpression efficiency of miR-138-5p in MCF-7 and MDA-MB-231 cells transfected with miR-138-5p mimics (miR-138-5p) or negative control miRNA mimics (miR-NC) was confirmed by qRT-PCR. **(C, D)** Dual luciferase reporter assay in MCF-7 and MDA-MB-231 cells co-transfected with microRNAs mimics (miR-NC or miR-138-5p) and psiCHECK2-containing-WT-LINC00467 or -Mut-LINC00467 vectors. Data were presented as the relative ratio of Renilla luciferase activity to Firefly luciferase activity. **(E, F)** Enrichment of LINC00467 and miR-138-5p in biotin-labeled anti-LINC00467 probes pull-down RNA fraction of MCF-7 and MDA-MB-231cells was determined by qRT-PCR. **(G, H)** Expression of miR-138-5p in LINC00467-depleted or -overexpressing MCF-7 and MDA-MB-231 cells was examined by qRT-PCR. Data are presented as mean ± S.D from three independent experiments. *P < 0.05; **P < 0.01. Student’s t-test.

We next explored the molecular mechanisms responsible for LINC00467 in progression of breast cancer carcinogenesis. Plenty of studies indicate LncRNAs take part in multiple process of tumorigenesis and cancer progression through suppressing miRNAs by acting as a “sponge” (9, 32, 33). We thereby searched for the downstream miRNA candidates of LINC00467. Bioinformatics analysis with miRcode algorithm (http://www.mircode.org/mircode/) showed miR-138-5p, which has been well known as a tumor suppressor in breast cancer (34, 35), was a potential miRNA target of LINC00467. To validate the facticity of this interaction, we first predicted the specific biding sites of miR-138-5p on LINC00467 transcripts using RNAhybrid (https://bibiserv.cebitec.uni-bielefeld.de/rnahybrid) and inserted full-length of LINC00467 transcripts containing wild-type or mutant miR-138-5p binding sites into the psiCHECK-2 vector (**Figure 4A**). Transfection efficiency of miR-138-5p mimics in MCF-7 and MDA-MB-231 cells was confirmed by qRT-PCR analysis (**Figure 4B**). Dual luciferase reporter assay indicated relative luciferase activity of wild-type LINC00467 transcripts was obviously impaired with the co-transfection of miR-138-5p, while the reporter constructs containing its mutant counterpart exhibited no difference in the luciferase activity when miR-138-5p was overexpressed (**Figures 4C, D**). Furthermore, a biotin RNA pull-down assay was performed to validate the direct interaction between LINC00467 and miR-138-5p. In line with what we expected, biotin-labeled probes against LINC00467 could significantly enriched endogenic LINC00467 and miR-138-5p in the pull-down fraction of MCF-7 and MDA-MB-231 cells (**Figures 4E, F**). Endogenous miR-138-5p levels were increased in LINC00467 silenced breast cancer cells (**Figure 4G**), and decreased with the ectopic expression of LINC00467 (**Figure 4H**). Collectively, these data suggested LINC00467 was physically associated with miR-138-5p and functioned as a “sponge” to suppress miR-138-5p expression.

### LINC00467 Associated With LIN28B to Up-Regulate Its Protein Level

**Figure 5:**
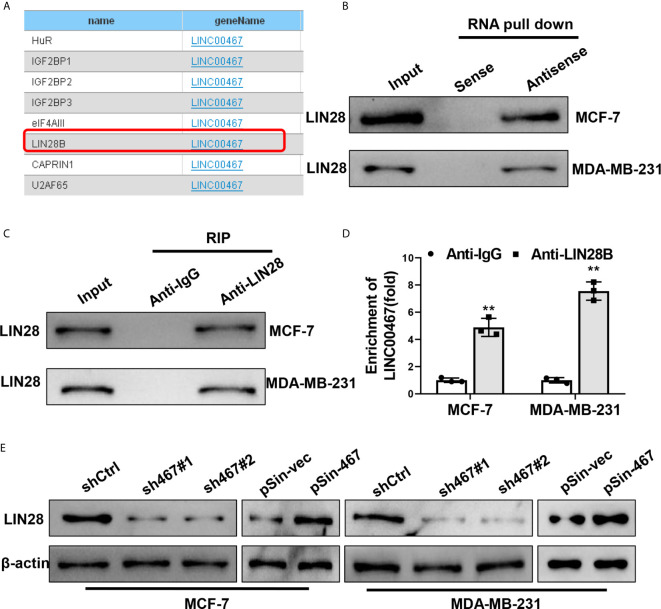
LINC00467 up-regulated LIN28B through a direct interaction. **(A)** Potential target RBPs of LINC00467 predicted by StarBase v2.0. **(B)** Biotinylated RNA pulldown assay showed the enrichment of LIN28B proteins in MCF-7 and MDA-MB-231 cell lysates pulled down by biotin-labeled LINC00467-antisense probes and biotin-labeled LINC00467-sense probes. **(C, D)** RNA immunoprecipitation assay showed the enrichment of LIN28B protein and LINC00467 in MCF-7 and MDA-MB-231 cell lysates by anti-LIN28B antibody and the control anti-IgG. **(E)** Western blot showed protein levels of LIN28B in MCF-7 and MDA-MB-231 cell with LINC00467 knockdown or overexpression. Data are presented as mean ± S.D from three independent experiments. **P < 0.01. Student’s t-test.

Numerous studies have reported LncRNAs’ functions in tumor progression depended on their interaction with RNA-binding proteins (RBPs) (7, 22). We also seek to identify the RBPs could physically interact with LINC00467 and mediate its oncogenic role in breast cancer progression. StarBase v2.0 (http://starbase.sysu.edu.cn/) was used to predict the potential RBPs targets that might be directly interacted with LINC00467 (**Figure 5A**), among which, LIN28B had been widely reported to be an important oncogene in aggressiveness of breast cancer (36, 37). We therefore focused on LIN28B to verify the interaction between each other. Biotin RNA pull-down assay showed that LIN28B could be notably enriched by biotin LINC00467-DNA-antisense probe as compared with biotin LINC00467-DNA-sense probe (**Figure 5B**). Reciprocally, anti-LIN28B antibody could also enrich LIN28B proteins (**Figure 5C**) and LINC00467 (**Figure 5D**), as determined by RNA immunoprecipitation (RIP) assay. Moreover, knockdown of LINC00467 reduced, and forced expression of LINC00467 increased protein levels of LIN28B in both MCF-7 and MDA-MB-231 cells, which was examined by western blot (**Figure 5E**). Taken together, these data indicated LINC00467 up-regulated the protein levels of LIN28B *via* a direct interaction in breast cancer cells.

### High Level of LINC00467 Correlated to Poor Prognosis of Breast Cancer Patients

**Figure 6:**
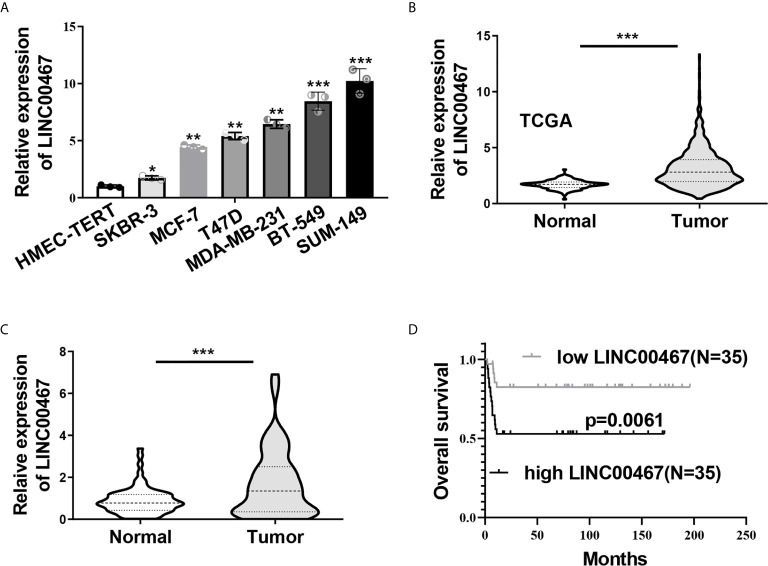
Increased LINC00467 predicted poor prognosis in breast cancer. **(A)** LINC00467expression in six breast cancer cell lines (SKBR-3, MCF-7, T47D, MDA-MB-231, BT-549 and SUM149) and a non-transformed mammary epithelial cell line (HMEC-HERT) was determined by qRT-PCR. **(B)** Expression level of LINC00467 in benign breast tissue samples (n=113) and breast cancer samples (n=1,091) in TCGA database. **(C)** qRT-PCR analysis of LINC00467 expression in 70 collected breast cancer specimens and paired adjacent normal tissues. **(D)** Kaplan-Meier analysis of the relationship between LINC00467 expression levels and overall survival of breast cancer patients. *P < 0.05; **P < 0.01; ***P < 0.001.

Last, we explored the clinical significance of LINC00467 in breast cancer. The expression of LINC00467 in a mammary epithelial cell lines (HMEC-HERT) and 6 breast cancer cell lines (SKBR-3, MCF-7, T47D, MDA-MB-231, BT-549 and SUM149) was first examined by qRT-PCR, a notably higher level of LINC00467 in breast cancer cell lines was observed compared to the non-transformed mammary epithelial cell line (**Figure 6A**). We then analyzed expression of LINC00467 in 113 normal breast tissues and 1,091 breast cancer samples from the TCGA datasets, which indicated LINC00467 was significantly overexpressed in breast cancer tissues as compared with their normal counterparts (**Figure 6B**). In consistence, qRT-PCR analyses of 70 collected breast cancer samples and matched adjacent samples demonstrated LINC00467 was remarkably up-regulated in breast cancer samples (**Figure 6C**). As showed in **Supplementary Table 2**, further analysis of correlation between LINC00467 expression and clinicopathological features of these enrolled breast cancer patients indicated LINC00467 expression was positively correlated with tumor stage (p=0.0164) and lymph node metastasis (p=0.0248) of breast cancer. Kaplan-Meier survival analysis revealed breast cancer patients with higher expression of LINC00467 exhibited a shorter overall survival (**Figure 6D**). Hence, these data suggested LINC00467 expression was increased in breast cancer and the upregulation of LINC00467 was associated with poor prognosis.

## Discussion

Despite it has been previously reported the oncogenic role in multiple cancer types (14–16, 18), the expression pattern and functional mechanism of LINC00467 in breast cancer has never been investigated. In the current study, we firstly reported that LINC00467 promoted proliferation, migration, invasion and EMT of breast cancer cells *in vitro* as well as tumor growth and lung metastasis in Balb/c nude mice. Further, LINC00467 inhibited the expression of a tumor suppressive miRNA: miR-138-5p, and up-regulated the protein level of an oncogene: LIN28B, *via* a direct interaction of each other. More importantly, elevated expression of LINC00467 was positively associated with lymph node metastasis and poorer overall survival of breast cancer patients, which emphasized the clinical significance of our research.

LINC00467, a greatly concerned LncRNA in cancer-related research recently, has been demonstrated to promote lung adenocarcinoma proliferation by sponging miR-20b-5p to activate CCND1 expression (38), regulate hepatocellular carcinoma progression by modulating miR-9-5p/PPARA expression (16). It has been increasingly reported competition with endogenous RNAs for the same miRNA binding sites was as a mode-of-action of lncRNAs (39). Here, we also found LINC00467 served as a miRNA “sponge” to suppress miR-138-5p expression. It has been demonstrated that miR-138-5p inhibited cell migration, invasion and EMT in breast cancer by directly targeting RHBDD1 (34), and repressed progression of breast cancer *via* NEAT1/miR-138-5p/ZFX axis (35), these previous results strongly indicated miR-138-5p was a tumor suppressor in breast cancer. We thereby believed LINC00467, at least partly, played the oncogenic role by inhibiting tumor-suppressive miR-138-5p expression in regulation of breast cancer progression.

Interaction with RNA biding proteins was another mode-of-action of lncRNAs. Just as reported, LINC00467 promoted lung adenocarcinoma proliferation, migration and invasion by binding with EZH2 and repressing HTRA3 expression (40). In addition, STAT1-induced upregulation of LINC00467 promotes the proliferation and migration of lung adenocarcinoma cells by epigenetically silencing DKK1 to activate Wnt/beta-catenin signaling pathway (41). In the present study, we successfully validated LINC00467 increased the protein level of LIN28B by directly interacting with each other. LIN28B has been broadly proved to play a critical oncogenic role in breast cancer. For example, it was reported LINC00665 promotes breast cancer progression through regulation of the miR-379-5p/LIN28B axis (36). Besides, LIN28B was also reported to promote proliferation of breast cancer cells *via* HBXIP-induced activation of TF II D (37). Given this, we suggested the enhanced effects of LINC00467 on the aggressiveness of breast cancer partly depended on the upregulation of protein level of LIN28B. However, how does LINC00467 increase the protein level of LIN28B with the interaction of each other, the specific mechanism involved remains to be further explored.

LINC00467 has been reported to be transcriptionally induced by STAT1 in lung adenocarcinoma cells (41); additionally, LINC00467 was up-regulated by TDG-mediated acetylation in non-small cell lung cancer (42). We had also tried to investigate the mechanisms responsible for the upregulation of LINC00467 in breast cancer. A number of famous cancer-related transcription factors such as STAT3, HIF-1α and MYC were chosen to study their effects on modulation of LINC00467 expression based on the prediction results of rVista 2.0 (https://rvista.dcode.org/instr_rVISTA.html). Not as expected, there was no obvious change in LINC00467 expression in breast cancer cells with the treatment of IL-6, hypoxia induction or silence of endogenic MYC. We speculated certain epigenetic-related regulatory mechanisms maybe involved in the process of LINC00467 upregulation; it would be interesting to figure this out in our further study.

In conclusion, our data indicated an involvement of aberrant LINC00467 overexpression in both proliferation, migration, invasion and EMT *in vitro* and tumor growth and lung metastasis *in vivo* of breast cancer cells, and uncovered the oncogenic properties of LINC00467 was attributed to LINC00467-mediated miR-138-5p inhibition and LIN28B up-regulation. Thereby, LINC00467 may serve as a potential therapeutic target in breast cancers.

## Data Availability Statement 

The datasets presented in this study can be found in online repositories. The names of the repository/repositories and accession number(s) can be found in the article/**Supplementary Material**.

## Ethics Statement

The studies involving human participants were reviewed and approved by Biomedical Ethics Committee of Anhui Medical University. The patients/participants provided their written informed consent to participate in this study. The animal study was reviewed and approved by Animal Ethical Committee of Anhui Medical University. Written informed consent was obtained from the individual(s) for the publication of any potentially identifiable images or data included in this article.

## Author Contributions

YZhu and YZha conceived and planned the study. YZha, YS and LD performed the experiments. KD helped to prepare and analyze the samples. YZha, YS, KD, WS and YZhu analyzed the data. YZhu and YZha wrote the manuscript. All authors contributed to the article and approved the submitted version. 

## Funding

This work was supported by Grants for Scientific Research of BSKY (XJ201912) from Anhui Medical University, the Scientific Research Foundation of Anhui Medical University (2019xkj010), The Natural Science Foundation of Anhui Province (2008085QH411).

## Conflict of Interest

The authors declare that the research was conducted in the absence of any commercial or financial relationships that could be construed as a potential conflict of interest.
