# Supplementary material for: Long Non-Coding RNA LINC00467 Correlates to Poor Prognosis and Aggressiveness of Breast Cancer
Source: Front Oncol. 2021 Apr 28;11:643394. doi: 10.3389/fonc.2021.643394 (PMC8113855; doi:10.3389/fonc.2021.643394)
Supplement: Supplementary file 1 [file Table_1.docx]

**Supplementary Table 1. Oligomers used in this study**

| Name | Application | Sequence |
| --- | --- | --- |
| qrt-LINC00467-F | qRT-PCR | CCTTCTTCCTCATCATCGTC |
| qrt-LINC00467-R | qRT-PCR | CCCAGTTTCAGTCCCTCTTG |
| qrt-GAPDH-F | qRT-PCR | TGCACCACCAACTGCTTAGC |
| qrt-GAPDH-R | qRT-PCR | GGCATGGACTGTGGTCATGAG |
| qrt-miR-138-5p-F | qRT-PCR | TGCAATGGGTTTGGCGTAGAAC |
| qrt-miR-138-5p-R | qRT-PCR | CCAGTGCCGCAGGGTAGGT |
| qrt-U6-F | qRT-PCR | CTCGCTTCGGCAGCACA |
| qrt-U6-R | qRT-PCR | AACGCTTCACGAATTTGCGT |
| LINC00467-shRNA1-F | plasmid construction | ccggGCTCTGTAAACCACATAATAAggatcc TTATTATGTGGTTTACAGAGCtttttg |
| LINC00467-shRNA1-R | plasmid construction | aattcaaaaaGCTCTGTAAACCACATAATAAggatccTTATTATGTGGTTTACAGAGC |
| LINC00467-shRNA2-F | plasmid construction | ccggGAAAACAAATAAATGGGAAAAggatccTTTTCCCATTTATTTGTTTTCtttttg |
| LINC00467-shRNA2-R | plasmid construction | aattcaaaaaGAAAACAAATAAATGGGAAAAggatccTTTTCCCATTTATTTGTTTTC |
| LINC00467-clone-F | plasmid construction | CTAGAATTCGTGGCGTAGGCCGGACATTT |
| LINC00467-clone-R | plasmid construction | CTAGGATCCAATTTCCAAACTCTTTATTATGTGG |
| LINC00467-clone for luci-F | plasmid construction | CTACTCGAGGTGGCGTAGGCCGGACATTT |
| LINC00467-clone for luci-R | plasmid construction | CTAGCGGCCGCAATTTCCAAACTCTTTATTATGTGG |
| LINC00467mut-clone for luci-F | plasmid construction | GCAACGAAAGAAGCACGAGGCGGCACGAA |
| LINC00467mut-clone for luci-R | plasmid construction | TTCGTGCCGCCTCGTGCTTCTTTCGTTGC |
| LINC00467-DNA-1-sense | lncRNA pull down | （biotin-）TGTCAAGTCGGACCAAACAGGTTGTTTCT |
| LINC00467-DNA-1-antisense | lncRNA pull down | （biotin-）AGAAACAACCTGTTTGGTCCGACTTGACA |
| LINC00467-DNA-2-sense | lncRNA pull down | （biotin-）CCAGACAGATTCAAGTATTGAAGATGCT |
| LINC00467-DNA-2-antisense | lncRNA pull down | （biotin-）AGCATCTTCAATACTTGAATCTGTCTGG |
| LINC00467-DNA-3-sense | lncRNA pull down | （biotin-）CCAGCACTGTGAAGCCTACAAAAACATT |
| LINC00467-DNA-3-antisense | lncRNA pull down | （biotin-）AATGTTTTTGTAGGCTTCACAGTGCTGG |

**Supplementary Table 2.** Correlation of LINC00467 expression with clinicopathological features in 70 breast cancer patients

| Expression of LINC00467 | | | |
| --- | --- | --- | --- |
| Variables Low (%) High (%) P value | | | |
| Age |  |  | 0.4667 |
| <50 | 13(44.83%) | 16(55.17%) |  |
| ≥50 | 22(53.66%) | 19(46.34%) |  |
| Triple-negative |  |  | 0.6313 |
| No | 15(46.88%) | 17(53.13%) |  |
| Yes | 20(52.63%) | 18(47.37%) |  |
| TNM staging |  |  | 0.0164* |
| Ⅰ-Ⅱ | 24(63.16%) | 14(36.84%) |  |
| Ⅲ-Ⅳ | 11(34.38%) | 21(65.63%) |  |
| Tumor size (cm) |  |  | 0.0557 |
| <5 | 21(61.76%) | 13(38.24%) |  |
| ≥5 | 14(38.89%) | 22(61.11%) |  |
| Lymph node metastasis |  |  | 0.0248* |
| Positive | 8(22.86%) | 17(48.57%) |  |
| Negative | 27(77.14%) | 18 (51.43%) |  |
